# Supplementary material for: Biophysical and biochemical evidence for the role of acetate kinases (AckAs) in an acetogenic pathway in pathogenic spirochetes
Source: PLoS One. 2025 Jan 9;20(1):e0312642. doi: 10.1371/journal.pone.0312642 (PMC11717252; doi:10.1371/journal.pone.0312642)
Supplement: S1 Fig — The AlphaFold2 model is shown in gray, and the Apo-TV0924 in varied colors (see Fig 4A in the main text). (PDF) [file pone.0312642.s001.pdf]

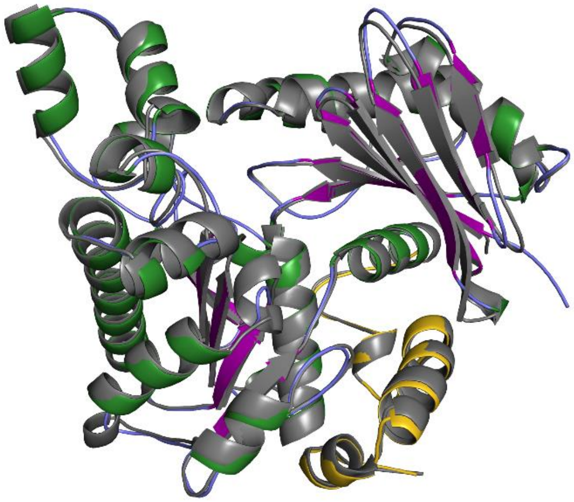

S1 Figure. Comparison of the final apo model to the AlphaFold2 model. The AlphaFold2 model is shown in gray, and the Apo-TV0924 in varied colors (see Fig. 4A in the main text).
