# Supplementary material for: Biophysical and biochemical evidence for the role of acetate kinases (AckAs) in an acetogenic pathway in pathogenic spirochetes
Source: PLoS One. 2025 Jan 9;20(1):e0312642. doi: 10.1371/journal.pone.0312642 (PMC11717252; doi:10.1371/journal.pone.0312642)
Supplement: S2 Fig — Mass photometry histograms shown at TV0924 concentrations of (A) 10 nM, (B) 20 nM, and (C) 30 nM. Blue histograms represent the number of contrast events per binned mass. Black lines are gaussian fits to the respective histogram peaks. (PDF) [file pone.0312642.s002.pdf]

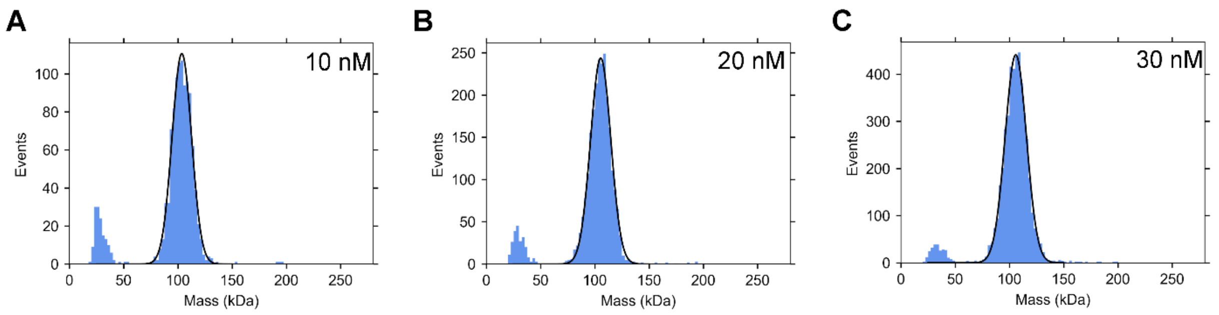

S2 Figure. Mass photometry of TV0924 after 63 h. Mass photometry histograms shown at TV0924 concentrations of (A) 10 nM, (B) 20 nM, and (C) 30 nM. Blue histograms represent the number of contrast events per binned mass. Black lines are gaussian fits to the respective histogram peaks.
