# Supplementary material for: Biophysical and biochemical evidence for the role of acetate kinases (AckAs) in an acetogenic pathway in pathogenic spirochetes
Source: PLoS One. 2025 Jan 9;20(1):e0312642. doi: 10.1371/journal.pone.0312642 (PMC11717252; doi:10.1371/journal.pone.0312642)
Supplement: S3 Fig — Shown are the refined positions of AMP and surrounding residues. Two electron-density maps are shown. The gray map shows an mFo−DFc map contoured at the 3-σ level of the omit map described in Fig 5, but without restricting the map to be close to the depicted atoms. The magenta map shows the same type of density contoured at the same level for the Apo-TV0924 structure. (PDF) [file pone.0312642.s003.pdf]

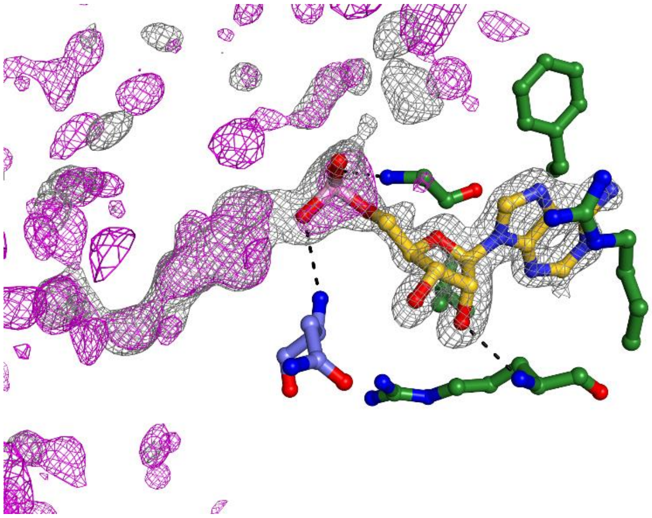

S3 Figure. Electron density in the active site of TV0924. Shown are the refined positions of AMP and surrounding residues. Two electron-density maps are shown. The *gray map* shows an $mF_o - DF_c$ map contoured at the 3-σ level of the omit map described in Fig. 5, but without restricting the map to be close to the depicted atoms. The *magenta map* shows the same type of density contoured at the same level for the Apo-TV0924 structure.
