# Supplementary material for: Biophysical and biochemical evidence for the role of acetate kinases (AckAs) in an acetogenic pathway in pathogenic spirochetes
Source: PLoS One. 2025 Jan 9;20(1):e0312642. doi: 10.1371/journal.pone.0312642 (PMC11717252; doi:10.1371/journal.pone.0312642)
Supplement: S4 Fig — Markers are the CD data monitored at 219 nm. Lines are fits to those data using (Eq 4) See inset legend for colors. (PDF) [file pone.0312642.s004.pdf]

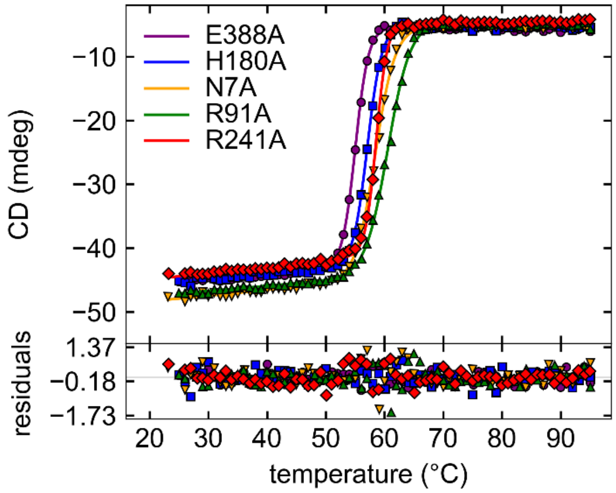

S4 Figure. CD-based melting curves for mutants of TV0924. Markers are the CD data monitored at 219 nm. Lines are fits to those data using Eq. 4 (see Main Text). See inset legend for colors.
