# Supplementary material for: Biophysical and biochemical evidence for the role of acetate kinases (AckAs) in an acetogenic pathway in pathogenic spirochetes
Source: PLoS One. 2025 Jan 9;20(1):e0312642. doi: 10.1371/journal.pone.0312642 (PMC11717252; doi:10.1371/journal.pone.0312642)
Supplement: S1 Table — All values are presented as the fitted Tm,app ± the 68.3% confidence interval. (PDF) [file pone.0312642.s005.pdf]

S1 Table. $T_{m,app}$ values for TV0924 mutants.

**Protein**	**$T_{m,app}$ (°C)**
N7A	58.48 ± 0.05[a]
R91A	60.61 ± 0.05
H180A	57.10 ± 0.03
R241A	58.60 ± 0.03
E388A	55.05 ± 0.02

[a]All values are presented as the fitted $T_{m,app}$ ± the 68.3% confidence interval.
